# Understanding statistical analysis in randomized trials: tips and tricks for effective review

**DOI:** 10.1093/ehjimp/qyaf036

**Published:** 2025-03-25

**Authors:** Paolo Frumento, Alessia Gimelli

**Affiliations:** Department of Political Sciences, University of Pisa, Via Serafini 3, 56126 Pisa, Italy; Department of Imaging, Fondazione Toscana Gabriele Monasterio, Via Moruzzi 1, 56124 Pisa, Italy

**Keywords:** statistical analysis, RCT, randomization

## Abstract

This review explores the critical role of statistical analysis in interpreting randomized controlled trials (RCTs), focusing on how these methods are used to evaluate the efficacy and safety of clinical interventions. RCTs are considered the gold standard in clinical research, yet their statistical complexity can make interpretation challenging. Understanding key statistical concepts, such as *P*-values, hazard ratios, and confidence intervals, is essential for distinguishing between statistical significance and clinical relevance. It is important to assess study design elements, including randomization methods, sample size calculations, and the handling of missing data, as these factors directly influence the validity of the findings. Additionally, the analysis plan—whether it follows an intention-to-treat approach or uses per-protocol analysis—can impact the interpretation of trial outcomes. Readers should also be aware of the distinction between pre-specified and *post hoc* analyses, as the latter can increase the risk of false positives. The appropriate interpretation of these elements ensures a balanced understanding of trial results, allowing clinicians and researchers to make evidence-based decisions.

## Introduction

Randomized controlled trials (RCTs) are often considered the gold standard for clinical research due to their ability to minimize bias and establish causal relationships between interventions and outcomes. However, reading and interpreting the statistical analysis of an RCT can be challenging, even for experienced readers. The analysis often involves complex statistical methods, with results presented using metrics like *P*-values, confidence intervals (CIs), hazard ratios (HRs), and more. This review provides a guide on how to read and interpret the statistical analysis of an RCT, highlighting key areas to evaluate, common pitfalls, and how to distinguish between statistical significance and clinical relevance. Relevant literature includes, among many others, Vijayananthan and Nawawi^1^ and Gamble *et al*.,^2^ to which we refer for further details.

## Key elements to focus on when reading an RCT

1. Study design and randomization
  - *Randomization method*: The quality of randomization is crucial in ensuring that the study groups are comparable. When reading an RCT, examine how randomization was performed—e.g. simple randomization, block randomization, or stratified randomization. Proper randomization minimizes selection bias. Example: In a cardiological trial using imaging endpoints, stratified randomization might be used based on factors like age, baseline left ventricular ejection fraction (LVEF), or presence of coronary artery disease to ensure balance in key clinical features across groups.
  - *Allocation concealment*: This process prevents foreknowledge of group assignment, reducing selection bias. Look for details about how allocation concealment was implemented—e.g. using opaque, sealed envelopes, or centralized randomization systems.
  - *Blinding*: Proper blinding (single, double, or even triple) helps prevent performance and detection biases. Ensure that the study describes who was blinded (participants, investigators, outcome assessors) and how blinding was maintained throughout the trial. Example: In a study assessing the effect of a new cardioprotective drug, blinding the physicians evaluating cardiac computed tomography (CT) scans can prevent subjective interpretations of changes in plaque volume or myocardial perfusion.
2. Sample size calculation and power analysis
  - Adequate sample size is essential for detecting a true effect if one exists. Check whether the authors provided a rationale for their chosen sample size, typically through a power analysis.
    Look for information about the anticipated effect size, variability of outcomes, and the significance level (*α*). If the study is underpowered (small sample size), it may fail to detect effects, leading to Type II errors (false negatives).
  - Conversely, studies with excessively large sample sizes may detect statistically significant differences that are not clinically relevant. Both statistical significance and clinical relevance of findings are important when interpreting study results.
3. Baseline characteristics
  - Examine the table of baseline characteristics to ensure that the groups are comparable at the start of the trial. This helps to confirm that randomization was effective. For example, severe patients may ‘hack’ the randomization process to be assigned to the treatment group.
  - In non-randomized studies, multivariable regression models or propensity score matching can be used to adjust for imbalance in baseline characteristics. In principle, no adjustments are necessary in RCTs, where observed and unobserved characteristics are expected to be balanced due to randomization and no confounding is present. However, in some cases, the randomization process might be partially unsuccessful, in particular, if the sample size is relatively small (e.g. in a subgroup analysis), making it necessary to apply to RCTs the same techniques designed for non-randomized studies.
4. Evaluating statistical analysis in RCTs
  4.1 Understanding the analysis plan
    - *Intention-to-treat (ITT) vs. per-protocol analysis*: ITT compares groups by treatment *assignment*, regardless of adherence to the intervention. It preserves the benefits of randomization and provides a conservative estimate of the treatment effect. Per-protocol analysis compares groups based on the received treatment, which may differ from the assigned one. Frequently, non-compliance occurs as some patients (typically the least severe) do not accept to undergo an experimental treatment. More complicated methods (e.g. principal stratification) may be used to address non-compliance.
    - *Pre-specified vs. post hoc analyses*: Results from pre-specified analyses carry more weight, as they minimize the risk of data dredging. Be cautious if the study relies heavily on *post hoc* analyses, as these can inflate the risk of Type I errors (false positives). While results based on pre-specified tests are seen as more reliable, *post hoc* findings are considered exploratory and hypothesis generating. This distinction is critical in avoiding the over-interpretation of results and ensuring that conclusions are grounded in rigorously planned analyses.
  4.2 Types of statistical tests
    - Understand the statistical tests used for comparing groups, which could include *t*-tests, *χ*^2^ tests, analysis of variance, or they non-parametric counterparts, as well as regression models.
    - Ensure that the choice of test is appropriate given the type of data (continuous vs. categorical) and the distribution of the data. For example, using a *t*-test assumes normally distributed data; if the data are very skewed or outliers are present, a non-parametric test like the Mann–Whitney *U* test might be more appropriate. For example, compare mean changes in LVEF between groups using *t*-tests or assess the association between treatment and improvement in coronary artery stenosis using regression models to adjust for potential confounders.
    - For time-to-event data, Kaplan–Meier curves and Cox proportional hazards models are commonly used. Example: In a trial evaluating the time to occurrence of major adverse cardiac events in patients receiving different interventions, Cox models might be employed, using changes in myocardial perfusion on nuclear imaging as a covariate.
  4.3 Adjustments for multiple comparisons
    - When a study performs multiple statistical tests, the risk of finding a false positive (Type I error) increases. To account for this, adjustments like the Bonferroni correction or false discovery rate control are commonly applied. For example, if a study compares changes in multiple cardiac magnetic resonance imaging–derived parameters (e.g. infarct size, myocardial strain, and ventricular volumes), adjustments for multiple comparisons can help to avoid spurious findings.
    - Another approach is to report a histogram of *P*-values or the proportion of *P*-values less than the significance level. For example, if no true association exists, the proportion of *P*-values <0.05 will be about 0.05. If much more than 5% of *P*-values are <0.05, this strongly suggests that some true associations may exist.
  4.4 Missing data and dropouts:
    Missing data are a common challenge in clinical trials. Examine how the authors dealt with missing data—e.g. through imputation methods, last observation carried forward (LOCF), or complete-case analysis.
    Assess the plausibility of different mechanisms of missingness (e.g. missing completely at random, missing at random, or missing not at random). Informative missing can introduce severe bias. For example, certain experiments can only be conducted on ‘sufficiently healthy’ patients, automatically excluding the most severe cases.
    The term ‘dropout’ refers to a particular missing data problem in which participants in a study leave the trial before its completion. Also in this case, it is important to assess whether the dropout occurred randomly or is correlated with individual observed characteristics or, in the worst-case scenario, depends on unobserved characteristics or potential outcomes. For example, patients who suffer from side effects of a drug might be incentivized to leave the experiment, while patients who have a better interaction with the treatment are more likely to remain.
    4.4.1 Types of missing data mechanisms
      - *Missing completely at random*: The probability of observing an individual is independent of all observed and unobserved characteristics. This scenario is less problematic as the missing data are effectively random.
      - *Missing at random*: Missingness depends on observed variables. Adjustments using statistical models (e.g. multiple imputations) can mitigate the bias.
      - *Missing not at random*: Missingness is related to unobserved outcomes (e.g. patients with a particular condition might prefer not to answer a questionnaire item regarding that condition). This scenario is the most challenging and can introduce substantial bias.
        Case (c) cannot be tested directly. In order to draw credible conclusions, plausible arguments should be made to assess the size and the direction of the bias. In some situations, the bias can be reasonably assumed to be negligible. Moreover, if the bias is most likely ‘towards the null’, a significant result provides even stronger evidence that a true association exists.

4.4.2 Handling missing data and dropout: It is possible to handle missing information in a number of ways:
  - *LOCF*: Assumes the last recorded outcome remains unchanged. This method is simple but can be unrealistic if outcomes are expected to change over time.
  - *Multiple imputation*: Predicts missing values based on available data and variability estimates. This method is more robust than LOCF but relies on appropriate model selection. Multiple imputation is particularly appropriate to ‘fill in’ the gaps in the predictors when the missing data occur sparsely.
  - *Inverse probability weighting*: Weights participants based on the likelihood of dropout to balance the comparison groups.

4.5 Crossovers:
  Crossover occurs when participants switch from their assigned treatment to the other study arm, either due to clinical necessity or due to patient preference. This phenomenon is particularly relevant in long-term trials and can dilute the estimated treatment effect.
  4.5.1 Types of crossovers
    - *Treatment crossovers*: Patients assigned to the control group switch to the active treatment (or vice versa). This often occurs in oncology or cardiac trials where ethical considerations permit switching to the effective treatment.
    - *Non-compliance-related crossovers*: Participants do not adhere to their assigned treatment but remain in the study.

4.5.2 Methods to handle crossovers
  - *ITT analysis*: ITT includes all patients in their originally assigned groups, regardless of crossover. While this preserves randomization, it may underestimate the true effect of treatment.
  - *As-treated analysis*: Groups participants based on the treatment they actually received. This approach may introduce bias since it disrupts randomization.
  - *Per-protocol analysis*: Includes only participants who adhered to their assigned treatment. While informative, this approach is prone to selection bias.
  - *Instrumental variable analysis*: A statistical method used to estimate the treatment effect while accounting for crossovers. It attempts to recover the causal effect by leveraging the random assignment as an instrumental variable.

5. Interpreting key statistical parameters
  5.1 The role of the *P*-value
    - *Significance threshold*: A *P*-value indicates the probability that the observed effect (or a more extreme one) could have occurred by chance if the null hypothesis is true. A common threshold for significance is 0.05 which, however, has no intrinsic property if not that of being a nice fraction in base 10. It is important to report the actual *P*-value and not just the binary classification ‘significant/not significant’.
    - *Limitations of P-values*: While a *P*-value can suggest whether an effect exists, it does not convey the magnitude or clinical relevance of that effect. A statistically significant result (e.g. *P* < 0.05) does not necessarily imply that the finding is clinically meaningful. For example, a statistically significant risk reduction might be too small to be considered clinically relevant or useful based on a cost–benefit analysis.
    - *Confidence intervals*: CIs provide a range within which the true effect size is likely to fall. A narrower CI indicates greater precision. Look for intervals that do not cross a null value (e.g. a risk difference of zero) to confirm statistical significance. For example, in a study reporting HRs for cardiac death based on echocardiographic findings, a narrower CI around the HR suggests more precise estimates.
  5.2 HR and its interpretation
    - *Definition*: A HR is a measure of relative risk in the context of time-to-event (survival) analysis. Unlike a simple relative risk (which compares cumulative event probabilities at a fixed time point), the HR specifically quantifies the rate at which an event occurs over time between two groups. An HR of 1 indicates no difference between groups, while a HR > 1 suggests a higher event rate in the treatment group compared with the control, and a HR < 1 suggests a lower event rate.
    - *Time dependency*: Because the HR represents a rate over time rather than a cumulative probability, it assumes that proportional hazards are the relative risk that remains constant throughout the study period. If this assumption is violated, alternative modelling approaches (e.g. time-varying effects or stratified Cox models) may be necessary. HR should always be reported with a CI to assess the precision and statistical significance of the estimate.
    - *Proportional hazards assumption*: For Cox regression models, the assumption of proportionality of hazards is critical. It assumes that the ratio of hazards between groups remains constant over time. Check whether this assumption is somewhat plausible (e.g. using Schoenfeld residuals). If not, one could divide the timeline in intervals and estimate a different HR in each interval.
  5.3 Effect sizes and clinical relevance
    - Look for effect size measures like Cohen’s *d*, odds ratios, or relative risk, which provide a sense of how strong the association is between the intervention and the outcome.
    - Evaluate whether the reported effect size has clinical significance. For example, a treatment that reduces the relative risk by 20% may sound significant, but if the absolute risk reduction is small, its real-world impact might be limited.
  5.4 The role of outcome selection in RCTs:
    The type and number of outcomes chosen for an RCT directly influence the study design, the statistical analysis, and its interpretability. In particular, distinguishing between primary, co-primary, and secondary outcomes is crucial for a comprehensive understanding of trial results.
    - *Primary vs. co-primary outcomes*: The primary outcome is the main endpoint used to determine the effectiveness of an intervention. Some studies define co-primary outcomes that represent additional endpoints to be met for the intervention to be considered successful. The use of co-primary outcomes can enhance the robustness of conclusions but requires careful statistical planning to avoid inflated Type I error rates.
    - *Composite outcomes*: Some trials use a composite primary outcome, which combines multiple individual endpoints into a single measure. While this approach can increase statistical power, it may introduce interpretability challenges, as not all components of the composite may be equally clinically relevant. Readers should examine whether all components contribute similarly to the overall effect and whether results are driven by a less meaningful endpoint.
    - *Multiplicity and statistical adjustments*: When multiple primary or secondary outcomes are analysed, the risk of false-positive findings increases. Methods such as Bonferroni correction or false discovery rate control can be applied to account for multiple comparisons and ensure robust conclusions.
    - *Patient-centred vs. surrogate endpoints*: Trials may use surrogate outcomes (e.g. biomarker levels and imaging findings) instead of patient-centred outcomes (e.g. survival and quality of life). While surrogate markers can provide early insights, their clinical significance should always be questioned.
  5.5 Hierarchical analysis of outcomes and the win ratio:
    In randomized controlled trials, hierarchical analysis of outcomes is used when multiple endpoints are of interest, and their relative importance follows a predefined ranking. This approach ensures that clinically meaningful outcomes are prioritized in the interpretation of results. One commonly used method for hierarchical analysis is the win ratio.
    - *Hierarchical analysis*: In trials employing hierarchical (or tiered) analysis, outcomes are assessed in a predefined sequence, ensuring that more critical endpoints (e.g. mortality) are analysed before less critical ones (e.g. symptom relief). This method avoids statistical issues associated with multiple comparisons and ensures that treatment effects are evaluated in a structured manner.
    - *The win ratio*: The win ratio is a statistical method used to compare treatment groups when multiple outcomes are ranked by clinical importance. Pairs of patients (one from the treatment group and one from the control group) are compared sequentially based on the hierarchy of outcomes. If a patient in the treatment group has a more favourable outcome on the highest-priority endpoint, this is considered a ‘win’. If the control patient has a better outcome, it is a ‘loss’. If there is no difference, the comparison moves to the next-ranked outcome. The win ratio is then calculated as the total number of wins divided by the total number of losses.
    - Advantages of the win ratio:
      - It provides a more nuanced comparison than traditional statistical tests, especially in trials with multiple relevant outcomes.
      - It allows for greater sensitivity in detecting treatment benefits by incorporating the clinical importance of each outcome.
      - It is particularly useful in cardiovascular and chronic disease trials, where composite outcomes are common, and mortality, hospitalizations, and symptom relief need to be weighed differently.

(d) Interpreting the win ratio
  - A win ratio greater than 1 suggests that the treatment is favoured over the control.
  - A win ratio close to 1 indicates little difference between groups.
  - CIs should be examined to assess the precision of the estimate, and *P*-values should be interpreted alongside clinical relevance.

6. Assessing data collection and sources of bias
  6.1 Quality of data collection
    - Evaluate how data were collected—e.g. self-reported outcomes, medical records, or direct measurements. Each method has different levels of reliability and susceptibility to bias.
    - Ensure that outcome measures are well-defined, validated, and standardized across all participants.
  6.2 Potential sources of bias
    - *Selection bias*: This can occur if randomization or allocation concealment is inadequate.
    - *Performance bias*: Can arise if participants or investigators are aware of the treatment assignment, leading to differential treatment beyond the intervention.
    - *Detection bias*: Occurs if outcome assessment is influenced by knowledge of group allocation.
    - *Attrition bias*: Results from differences in withdrawal rates between groups. Look at how many participants dropped out and why.
  6.3 Subgroup analyses
    - Subgroup analyses can provide insights into how different populations respond to the intervention. However, they can be prone to over-interpretation.
    - Be cautious with subgroup findings, especially if they were not pre-specified, as these can result in spurious associations due to reduced sample sizes and multiple comparisons.
7. Common pitfalls in interpretation
  7.1 Confounding factors
    - Look for potential confounders that could explain the observed effect. If confounding is likely, the study should adjust for these variables using regression analysis or stratification. For example, in a study evaluating a new lipid-lowering drug's impact on plaque regression seen on coronary CT, potential confounders like baseline lipid levels and the use of concomitant statins should be accounted for in the analysis.
    - Unadjusted results can be misleading, making it important to assess both crude and adjusted analyses.
  7.2 Overemphasis on statistical significance
    - A common error is to place too much emphasis on the *P*-value while neglecting effect sizes, CIs, and clinical significance.
    - Consider the overall body of evidence rather than relying solely on whether results reach statistical significance.
  7.3 Generalizability of results
    - Consider whether the trial population is representative of the broader population to which the results will be applied. Factors such as strict inclusion/exclusion criteria can limit generalizability. For example, a trial that excludes patients with severe renal impairment due to the risk of contrast use in cardiac CT might limit the generalizability of the results to a real-world population with a high burden of comorbidities.
    - Evaluate the trial setting, duration of follow-up, and nature of the intervention to determine whether the results are applicable in real-world practice.

## Conclusion

Reading and interpreting the statistical analysis of an RCT involves a careful balance between understanding the methods, evaluating the robustness of the analysis, and assessing the clinical relevance of the findings. By focusing on key areas like randomization, sample size, types of analyses, and interpretation of statistical metrics like *P*-values and HRs, one can gain a clearer understanding of the study’s validity and applicability. Remember that while statistical significance is important, it is not synonymous with clinical significance, and the true impact of a study’s findings often lies in the details beyond the *P*-value.

## Data Availability

No new data were generated or analysed in support of this research.

## Lead author biography

Paolo Frumento is currently a professor of statistics at the Department of Political Sciences, University of Pisa. Previously, he has been an assistant professor at Karolinska Institute, Stockholm, Sweden. His main areas of expertise include statistical modelling, regression analysis, survival analysis, and quantile regression.
